# Epidemiology of persistent iatrogenic spinal cord injuries in Western Norway

**DOI:** 10.1002/brb3.522

**Published:** 2016-07-07

**Authors:** Mathias S. Æsøy, Stein‐Erik H. Solvang, Marit Grønning, Tiina Rekand

**Affiliations:** ^1^ Department of Clinical MedicineUniversity of BergenBergenNorway; ^2^ Department of Occupational MedicineHaukeland University HospitalBergenNorway; ^3^ Department of NeurologyHaukeland University HospitalBergenNorway; ^4^ Institute of Neuroscience and PhysiologySahlgrenska AcademyUniversity of GothenburgGothenburgSweden

**Keywords:** complication, epidemiology, iatrogenic, injury, neurological disorders, spinal cord injury

## Abstract

**Objectives:**

Iatrogenic spinal cord injuries (SCIs) caused by invasive procedures or surgical interventions have previously been reported as case studies. The primary objective of this study was to investigate and analyze the incidence, etiology, and prognosis of iatrogenic SCI in Western Norway.

**Methods:**

Medical records of all 183 patients admitted to the SCU between 01.01.2004 and 31.12.2013 were reviewed. Gender, age, diagnosis, iatrogenic medical procedure, symptoms and findings before and after injury, mechanism of injury, level of injury, and ASIA Impairment Scale (AIS) score prior iatrogenic SCI, at admittance and discharge were recorded, as were the length of the period prior to admittance and the length of stay.

**Results:**

Twenty‐three (12.5%; 14 men, nine women) of 183 patients met the criteria for iatrogenic SCI. The annual incidence rate was estimated 2,3 per 1,000,000 (*SD* ±1.0). Mean age at iatrogenic SCI was 55.5 years (range 16–79 years). Intervention for cervical spinal stenosis was the leading cause of iatrogenic SCI, followed by operations on the aorta and spine. Iatrogenic SCIs was most frequently located on the thoracic level. The patients suffered from clinical incomplete injuries (AIS score C and D) both at admittance and discharge from the SCU. Most patients improved, but no patient recovered completely after SCI.

**Conclusion:**

Although the annual incidence rate of iatrogenic SCI is low in Norway, individual consequences are serious. Increased awareness of the causes of SCI may decrease the risk of iatrogenic SCI.

## 1 Introduction

*Primum non nocere*, the Latin for *above all, do no harm*, is a fundamental principle in medicine. The word *iatrogenesis* (from the Greek) translates into *brought forth by the healer* and is defined as any adverse effect or condition occurring as a result of medical treatment, investigation, or advice.

Spinal cord injuries (SCIs) may cause variable degrees of motor, sensory, and/or autonomic deficits. The definition of SCI includes the cauda equina, but isolated root injuries are excluded from this definition (Hagen et al., 2010, 2012). In general, a SCI can arise from various causes, including iatrogenic events. Several isolated cases and interventions leading to iatrogenic SCI have been previously described. A previous study from Spain showed that 14.7% of patients with spinal cord injuries had iatrogenic origin (Alcanyis‐Alberola et al., 2011). Iatrogenic SCIs caused by surgical interventions have otherwise previously been reported mainly as case studies. Epidemiological studies are needed to evaluate the scope of this problem. Such spinal cord injuries might be preventable.

The primary objective of this study was to investigate and analyze the epidemiology of iatrogenic SCI over a period of 10 years, including the annual incidence rate, causes, mechanism of injury, level, and prognoses of iatrogenic SCI.

## 2 Materials and Methods

**Figure 1:**
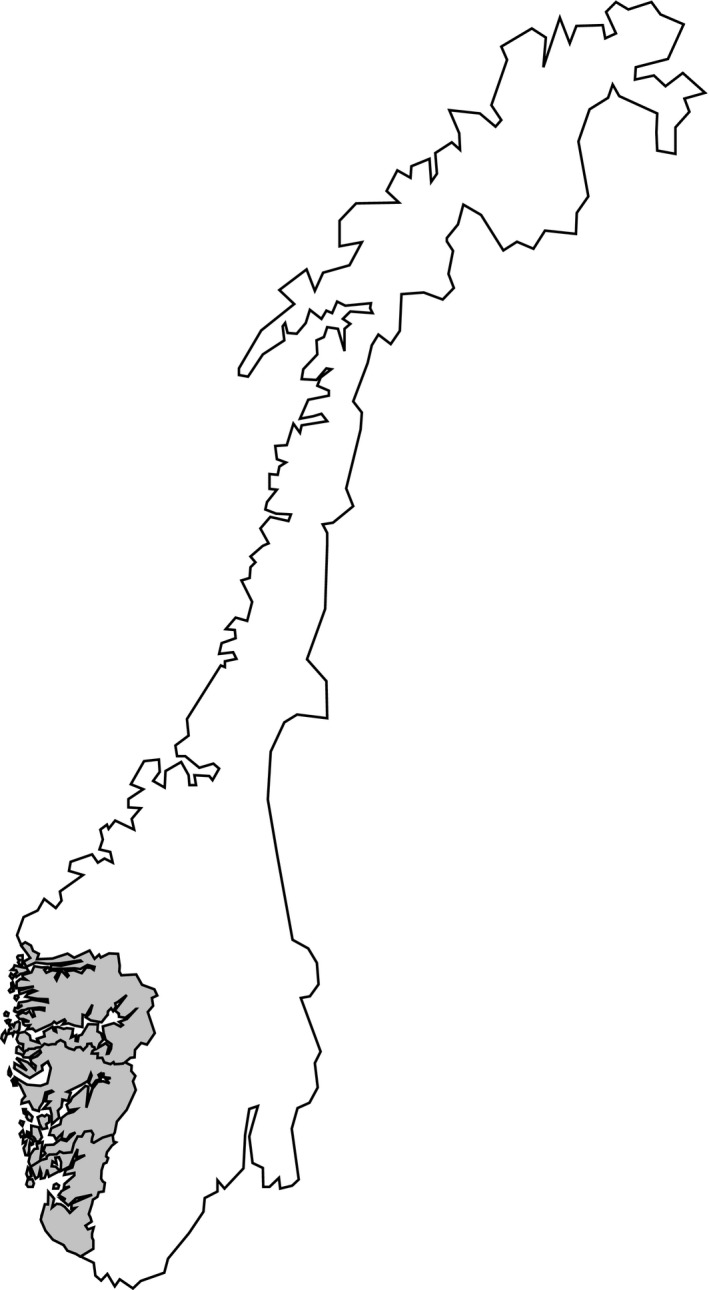
Map of Norway with marked study area

Rehabilitation of spinal cord injuries are divided between three spinal cord units in Norway. Primary rehabilitation of spinal cord injuries will not be performed in other hospitals and follow‐up will be performed either at spinal cord units or under supervision of spinal cord units. The spinal cord unit (SCU) at Haukeland University Hospital, Bergen, is the only SCU in Western Norway. This SCU has regional responsibility for the management, treatment, and rehabilitation of all patients with SCI in three counties in Western Norway (Fig. 1). The spinal cord unit has 12 beds. There are no other rehabilitation facilities in Western Norway with competence or facilities to provide primary rehabilitation after spinal cord injury. The medical records of all patients admitted with acute SCI to this SCU from 01.01.2004 to 31.12.2013 were reviewed.

Patients were eligible for inclusion in this study if they had a SCI that developed after diagnostic procedures or treatment. In one case, the patient's bladder dysfunction developed following the operation on spinal tumor, septic complications and birthgiving. The bladder was permanently catheterized during the operation, following complications and procedures. During the rehabilitation, the bladder dysfunction was clinically demonstrated, but exact cause remained unclear. The patient was not included in this study.

For patients with iatrogenic SCI, the following variables were collected: gender, age at the time of injury, diagnosis that led to the intervention, iatrogenic medical procedure, mechanism of injury, level of injury, symptoms and findings before and after SCI, American Spinal Injury Association Impairment Scale (AIS) score at admittance and at discharge (http://www.asia-spinalinjury.org/elearning-workshett-2015-web.pdf. Accessed March 29, 2016), number of days before admittance to the SCU, and length of stay in the SCU. The American Spinal Injury Association (ASIA) Impairment scale ranges the completeness of spinal cord injury between A (complete spinal cord injury) to E (injury has caused no permanent neurological findings).

We estimated the annual incidence of iatrogenic SCI by extrapolating the number of iatrogenic SCIs in our sample to the population of Western Norway in the study period. Mean annual number of inhabitants was 993,341, the number of inhabitants was stable during the observation period Standard deviation was calculated. To calculate the proportion of iatrogenic SCIs among all SCIs, we divided the number of iatrogenic SCIs by the total number of patients admitted to the SCU during the study period. Information was systematized and various data were compared. The chi‐squared test was used to determine significant differences between groups, and Student's *t* test was used when variables were continuous. Significance was set at *p* < .05.

The study was retrospective and did not include any interventions. The project was discussed with privacy ombudsman at Haukeland University Hospital, who concluded that an approval by the Regional committee for medical and health research ethics was not needed.

## 3 Results

**Figure 2:**
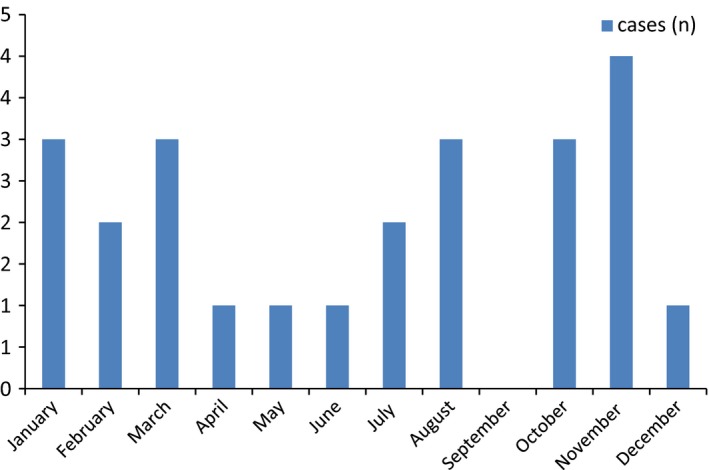
Number of observed iatrogenic SCI cases per month 2004‐2013

**Figure 3:**
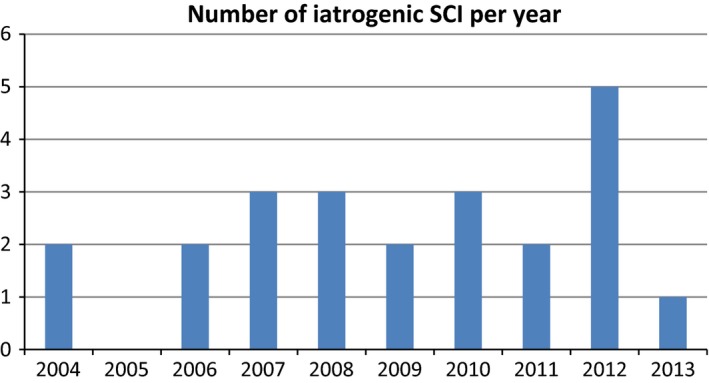
Number of iatrogenic SCI every year 2004–2013

A total of 183 patients were admitted to the SCU during the study period. Twenty‐three patients (12.5%) met the criteria for iatrogenic SCI, yielding a yearly incidence of iatrogenic SCI of 2.3 per 1,000,000. During the observation period iatrogenic spinal cord injuries occurred in all months except September and every year except 2005 (Figs 2, 3). Among all patients with iatrogenic SCI, 14 patients were male (60.9%) and nine patients were female (39.1%, *p* = .069). The average age at the time of injury was 55.5 years. The patients were hospitalized to the SCU in average after 49 days (range 7–334 days) and the average duration of stay was 90 days (range 10–177 days).

Table 1 presents patient diagnoses, procedures, causes of injury, and symptoms and findings before and after injury. Eleven patients (47.8%) displayed no neurological deficits prior to their procedures. In most patients varying degrees of radicular distribution of pain or motor impairment, bowel and urological dysfunction as consequences of SCI were observed. All patients suffered from clinically incomplete SCI after the procedure. Seventeen patients (73.9%) underwent procedures directly involving the spine; thus the operation most probably was a main cause for iatrogenic SCI. Intervention for cervical spinal stenosis was the most frequent cause of iatrogenic SCI (21.7%; Table 1). Patients in this group developed cervical lesions with tetraplegia. These were the oldest group in our study, with an average age of 67.2 years. Patients who underwent surgery for scoliosis developed thoracic lesions and were the youngest patient group (average age 29 year). Various interventions caused injury by different mechanisms. For example, degenerative spinal disease predominantly caused SCI due to direct manipulation of the spinal cord, while intervention for aortic aneurysm caused SCI via ischemic infarction.

**Table 1:** Procedures, causes and symptoms related to SCI

Diagnosis (*n*)	Procedure (*n*)	Cause of injury (*n*)	Symptoms and findings before procedure (*n*)	Symptoms and findings after injury (*n*)
Spinal stenosis (5)	Laminectomy (4)Discectomy (2) Disk prosthesis (1)	Manipulation of spinal cord (4) Epidural hematoma (1)	Pain (4) Motor impairment (3) Sensory impairment (5)	Pain (3) Motor impairment (5) Sensory impairment (2) Bladder dysfunction (3) Bowel dysfunction (3)
Aortic aneurysm (4)	Aortic resection (4)	Infarction (4)	No neurological findings (4)	Pain (3) Motor impairment (4) Sensory impairment (2) Bladder dysfunction (2) Bowel dysfunction (4)
Scoliosis (4)	Correction of scoliosis (4)	Manipulation of spinal cord (1) Infarction (3)	No neurology (3) Pain (1) Sensory impairment (1)	Pain (1) Motor impairment (4) Sensory impairment (3) Bladder dysfunction(3) Bowel dysfunction (1)
Prolapsed disk (3)	Laminectomy (1) Discectomy (1) Disk prosthesis (1) Microsurgical extirpation (1)	Manipulation of spinal cord (3)	Pain (2) Motor impairment (2) Sensory impairment (2)	Pain (2) Motor impairment (2) Sensory impairment (1) Bladder dysfunction (2) Bowel dysfunction (2)
Spondylodiscitis (2)	Laminectomy (1) Fixation (1)	Manipulation of spinal cord (1) Epidural hematoma (1)	Pain (2) Motor impairment (1)	Pain (2) Motor impairment (2) Bladder dysfunction (1)
Cancer (2)	Lumbar puncture with anesthesia (1) Biopsy of lung tumor (1)	Subdural hemorrhage (1) Air emboli in spinal cord—infarction (1)	No neurological findings (2)	Motor impairment (2) Sensory impairment (2) Bladder dysfunction (2) Bowel dysfunction (1)
Knee ligament injury (1)	Lumbar puncture with anesthesia	Subdural hemorrhage	No neurological findings	Sensory impairmentBladder dysfunction (1) Bowel dysfunction (1)
Subdural cyst (1)	Resection of cyst	Epidural hemorrhage	Motor impairment Sensory impairment Bladder dysfunction	PainMotor impairmentSensory impairment Bladder dysfunctionBowel dysfunction
Delirium (1)	Diagnostic lumbar puncture	Epidural hemorrhage	No neurological findings	Motor ImpairmentBladder dysfunctionBowel dysfunction
Chronic pain after whiplash (1)	Radiofrequency ablation	Thermal injury of spinal cord	PainSensory impairment	Pain ParesisSensory impairmentBladder dysfunctionBowel dysfunction
Spondylosis (1)	Decompression and fusion	Manipulation of spinal cord	Pain Motor impairment	Pain Motor impairment Sensory impairment

All patients suffered more pronounced loss of neurological function immediately after procedures (Table 1).

Injury levels and AIS score prior surgery or invasive procedure, upon admission and discharge are shown in Table 2. Five patients had spinal cord injury (AIS D) before iatrogenic SCI. All patients suffered from either deterioration of SCI measured by AIS or developed SCI as a consequence of surgery or invasive procedure (*p* = .001). Injury levels significantly differed according to diagnoses and the anatomical region of the procedure. The majority of patients improved their daily function during the hospital stay although only six patients (26.1%) significantly achieved improved AIS scores upon discharge. These patients were admitted to the SCU an average of 36 days earlier, and stayed for 37.5 days longer on average, than patients whose AIS scores were not improved at discharge.

**Table 2:** Level of injury and impairment score (AIS) prior surgery or invasive procedure at admission and discharge from the SCU

Level of disease	AIS prior surgery or invasive procedure (*n*)	AIS at admission (*n*)	AIS at discharge (*n*)
Cervikal (8)	D (3)	B (2)	B (2)
		C (3)	C (1)
		D (3)	D (5)
Thoracic (9)	D (1)	B (1)	B (1)
		C (6)	C (6)
		D (2)	D (2)
Lumbar (6)	D (1)	C (3)	C (2)
		D (3)	D(4)
Total	D (5)	B (3)	B (3)
		C (12)	C (9)
		D (8)	D (11)

## 4 Discussion

In this study, iatrogenic SCI comprised 12.5% of all SCIs admitted to the SCU during the study period. This is the first epidemiological study of iatrogenic SCI in Norway. Our study revealed that iatrogenic spinal cord injuries occurred nearly every year and there were no differences on monthly basis. Only few publications have addressed the epidemiology of iatrogenic SCI in general. Alcanyis‐Alberola et al. (2011) reported that iatrogenic SCI comprised 10.4% (26/250) of all patients admitted during a 4‐year period in Valencia, Spain. This incidence is close to our calculated rate, and supports the need for expanded awareness of iatrogenic SCI.

Traumatic SCI in everyday life is mainly caused by accidents (Hagen et al., 2012). In a previous study of traumatic SCI in the same area as this study, the calculated incidence was up to 21.2/1,000,000 (Hagen et al., 2010); 41.4% of the cases suffered from complete injuries, the average age at injury was 42.9 years, and the injured were most frequently male (Alcanyis‐Alberola et al., 2011). In this study, no patients suffered from complete injury. In the study of Alcanyis‐Alberola et al. (2011) 91% of the patients with iatrogenic SCI suffered from in incomplete SCI. The patients with iatrogenic SCI seem to be older and less injured than patients with traumatic SCI. Hagen and coworkers also reported an increasing incidence of traumatic SCI among the elderly (Hagen et al., 2010). As the general population ages, this increase in age may also occur for iatrogenic SCI, a possibility that is also underscored by the observation that old age is associated with increases in various comorbidities which may complicate procedures (Chen, Li, & Wu, 2012; Martin et al., 2005). Preventive measures such as the development of guidelines and good monitoring during procedures are therefore of paramount importance in order to avoid an increase in the occurrence of iatrogenic SCI.

In the current investigation degenerative spine disease, aortic aneurysm, scoliosis, and spondylodiscitis were the most frequent diagnoses related to procedures that caused SCI (73.9%). Interventions for these conditions are known to be associated with risk for SCI (Amiri et al., 2013; Bacher et al., 1999; Chen et al., 2012; Piffaretti et al., 2014; Tanaka et al., 2014; Vitale et al., 2010). For example, in 2014, Tanaka and coworkers reported that 3.5% of patients who underwent an operation for aortic aneurysm suffered postoperative SCI (Tanaka et al., 2014).Fortunately, preventive techniques and monitoring reduce the risk of SCI during these procedures (Devlin & Schwartz, 2007; Pastorelli et al., 2011; Shimizu & Yozu, 2011; Tanaka et al., 2014).

Monitoring of the spinal cord has been developed for early detection of neurological complications during surgery for spinal deformities (Bacher et al., 1999). Combined use of both sensory‐evoked potential and transcranial motor‐evoked potential provides the opportunity to monitor the major medullary pathways and the central part of the spinal cord (Vitale et al., 2010). Correction of spinal deformity is considered to be the most dangerous stage of scoliosis surgery (Pastorelli et al., 2011). Surveillance enables the surgeon to reverse maneuvers that lead to a fall in motor‐evoked potential and/or sensory‐evoked potential and to avoid incidents that may have developed into SCIs (Pastorelli et al., 2011). In our study cohort, none of the patients with scoliosis and iatrogenic SCI had pertinent neurological findings before SCI, and they were not monitored during the operation. All patients had complicated and pronounced deformity of the spine. However, monitoring the spinal cord during this type of procedure is now standard at our hospital.

Spinal ischemia is a known complication of aortic surgery. In our study, all patients with aortic disease underwent surgery in an emergency situation. One patient received treatment for known high blood pressure, two patients were treated for elevated blood pressure during the operation, and one patient suffered cardiac arrest before surgery. All patients had unstable hemodynamics. The need for acute intervention in these cases may therefore make complications like SCI less preventable.

Operations on anatomically challenging cases and treatment of patients at high risk for complications increase the incidence of iatrogenic injuries (Chen et al., 2012). Thorough decision making to identify an effective strategy before surgery is therefore critical. Procedures are complex and often must tackle several complicating factors such as high age, hypertension, preoperative spinal cord injury (Piffaretti et al., 2014; Uchida, 2014).Our patients with SCI were older than other patient groups and suffered several comorbidities.

Three patients developed SCI after spinal anesthesia or lumbar puncture because local or general guidelines for the procedures were not followed. One lumbar puncture led to an epidural hematoma because the doctor was not aware that the patient received anticoagulant treatment before the procedure. The other two subdural bleeds resulted from multiple puncture attempts at aberrant spine levels. In these three cases, diagnoses of complications were delayed due to lack of clinical follow‐up after the procedures.

In one patient SCI after radiofrequency ablation was caused by a misplaced needle that caused direct thermal damage to the spinal cord. In another case, lung‐tumor biopsy was performed twice due to lack of communication between two hospitals. The second biopsy launched an air embolism into the spinal cord. These patients had minor or no neurological deficits prior to injury, and SCI should have been avoided.

The patients in our study suffered from incomplete SCI. Although AIS score improved in only six patients after rehabilitation, the majority of patients improved their daily function. The AIS score is not sensitive enough to detect all minor but still individually significant improvements in function. Patients in the study of Alcanyis‐Alberola et al. (2011) also had AIS score improvements similar to the improvements in our patients. Taken together, these findings imply that recovery from iatrogenic SCI follows the same pattern as other traumatic incomplete SCIs (Waters et al., 1994).

The study has some limitations. This study was retrospective and included the patients who had persistent spinal cord injuries. Transient spinal cord injuries have not been mapped in this study. The patients with terminal metastatic cancer with life expectancy shorter than 6 months are likely not to be referred to rehabilitation. Although the SCU at Haukeland University Hospital is responsible for the rehabilitation of all patients with SCIs in the region, there is a slight chance that some patients with minor nonmotor injuries were treated elsewhere. For example, mild urological dysfunction following a small lesion of the conus medullaris may not been diagnosed as a consequence of iatrogenic SCI.

In conclusion, our study revealed that a little more than two patients develop SCI as a consequence of medical procedure in Western Norway every year. The causes of iatrogenic SCI differ, but the most frequent causes in Western Norway are surgical interventions for spinal deformities and diseases. The procedures and causes of iatrogenic injuries probably have similarities worldwide. The current investigation may therefore increase awareness of potentially harmful procedures and consequently prevent SCI.

## Funding Information

No funding information provided.
